# The Development of Criteria for the Selection of Chinese Peer Educators in HIV Management: A Delphi Study

**DOI:** 10.1155/2016/9521313

**Published:** 2016-11-24

**Authors:** Yan Qiu, Jiayin Shen, Hongzhou Lu

**Affiliations:** ^1^Shanghai Public Health Clinical Center, Fudan University, Shanghai 201508, China; ^2^School of Nursing, Fudan University, Shanghai 200000, China

## Abstract

To develop an expert consensus opinion of the criteria for selection of peer educator for HIV/AIDS care program, twenty experts were invited to complete a two-round Delphi consultation. Response rates of the panelists for the first and second rounds were both 100%, and the authority coefficient of the experts was 0.75. Experts achieved consensus on a total of nine items (75%) on completion of the study. The Precedence Chart Method was used to calculate the weight of each indicator, and then a priority list was generated accordingly. This study provides the initial selection criteria for HIV/AIDS peer educators in Shanghai as well as in China.

## 1. Introduction

Among the reported 610,903 people living with HIV/AIDS in China by the year of 2016, more than 50,000 were newly infected in the first half of 2016 [1]. However, medical staff caring for HIV-infected patients was not increased accordingly, which resulted in a shortage of trained medical staff. At the same time, World Health Organization (WHO) and The Joint United Nations Programme on HIV and AIDS (UNAIDS) have defined that the lack of the human resources is the biggest obstacle to expand medical services [2]. To address this continuing increase in the demand for HIV medical services, several measures have been proposed including recruiting healthcare workers from the non-AIDS clinics where human resources are relatively rich as well as training nonprofessional healthcare staff [2, 3].

Actually, community based, nonprofessional caregivers have become the main human resources in the HIV medical service team in many countries [4–6]. Notably, numerous reports have demonstrated that supports from HIV-infected patients (peers) themselves could improve the antiretroviral therapy compliance, reduce HIV related stigma, and improve the safety and quality of patients' life [4–12].

It has been revealed that peer educator is the key factor directly related to the outcome of peer education. Therefore, selecting peer educators is one of the most fundamental factors determining the success of peer support. Undoubtedly, being infected with HIV is a prerequisite for being a peer educator, but it does not mean that any HIV-infected person could be a qualified peer [7, 13]. Although several important characteristics and guidelines have been proposed to be considered while selecting HIV peer educators, it is believed that accommodation of the potential needs of the given group of HIV-infected people is crucial for creating an effective selection strategy. Unfortunately, there is no such norm or guideline for selecting HIV peer educators hitherto since peer education for HIV infection management is still in its infancy in China.

In order to implement the HIV peer education in our hospital, the initial step is to develop clear criteria for the selection of peer educators. Delphi, an approach to achieve a unified opinion through a number of rounds of consultation with experts' advice and feedback on a subject or matter, is widely employed by nursing research [14–16]. Therefore, Delphi method is adopted in this study to fulfill our needs.

## 2. Materials and Methods

A modified Delphi study with 2 phases as described before was performed [14–16]. This study was approved by the Shanghai Public Health Clinical Center Ethics Committee. Confidentiality and anonymity of the participants were secured.

Potential indicators were selected through a systematic review of the relevant literature. Keywords including “peer supporter”, “social worker”, “community health worker”, “chronic disease”, and “training program” were used to search the databases such as “The Cochrane Library,” “JBI,” “PubMed,” and “Embase,” as well as “The Joanna Briggs Institute Library.” Ten most relevant articles were included after extensive review of the titles, abstracts, and main descriptions. After a further round of review by ten experts according to the criteria published by the JBI Health Center, one paper with low quality was excluded. Data were extracted from the identified articles, and 10 selection/recruitment standards were chosen to create the primary questionnaire based on experts' consensus.

### 2.1. Delphi Panel

All experts who participated in this study have extensive experiences in the HIV treatment and clinical care. In the first round, questionnaires were delivered to the experts individually by email in March 2016, and responses were collected within two weeks. Completed questionnaires were analyzed, and then a modified questionnaire was formed based on experts' agreement. There was one-week interval before the initiation of the second round. Responses for each item of the questionnaire were evaluated by using the five-point Likert scale (extremely important, important, moderately important, little important, and unimportant). In this study, the consensus was achieved after two rounds.

### 2.2. Statistical Analysis

The ratio of the number of questionnaires delivered to the number of those returned was calculated as an indicator of experts' enthusiasm. The authority coefficient (*C*_*r*_) was used to evaluate the authority degree of experts, which is determined by two factors: the basis of judgment (*C*_*a*_) and familiarity of the question (*C*_*s*_). The Cr is defined as *C*_*r*_ = (*C*_*a*_ + *C*_*s*_)/2, and the acceptable reliability level is *C*_*r*_ ≥ 0.7.

In the first round of the Delphi procedure, the response of each item was rated using Likert-type scale items: 5 (*extremely important*), 4 (*important*), 3 (*moderately important*), 2 (*less important*), and 1 (*unimportant*). A descriptive analysis of the 10 items that reached consensus of being important (items were viewed as being “extremely important” or “important” by at least 80% of respondents) was summarized. In the second round, the Precedence Chart Method was adopted to calculate the index weight of each indicator. The consistency between the expert opinions was determined using Kendall's W.

## 3. Results

### 3.1. The Characteristics of the Experts

As shown in Table 1, of the 20 experts 13 (65%) were females and 7 (35%) were males. Most experts (19, 95%) who participated in this study had a bachelor's degree or higher. All participants were affiliated with a university hospital. Fifty-five percent (11/20) of the experts were nurses, while the rest were doctors. Moreover, eighty-five percent (17/20) of the experts had work experiences in the field of HIV infection prevention and treatment.

Experts' enthusiasm reached 100% as all of the twenty experts responded timely for the two rounds of consultations. Seven (35%) experts believed that they were extremely familiar with the question, and half (10/20) of the experts thought that they were familiar with the question. Notably, all of the expert's Cr (coefficient of expert's authority) was above 0.75 (*C*_*r*_ = 1.0, *n* = 4; *C*_*r*_ = 0.95, *n* = 6; *C*_*r*_ = 0.9, *n* = 6; *C*_*r*_ = 0.85, *n* = 2; *C*_*r*_ = 0.75, *n* = 2), so the general opinion of expert advice is acceptable.

### 3.2. The First Round

Ten primarily extracted indicators were evaluated by the expert panel in the first round of the Delphi procedure, and experts of this panel reached consensus on five items (50%). Notably, all the items which achieved consensus were viewed as “extremely important” or “important.” Items that achieved a complete consensus were “individuals with good self-management skills for HIV” (100%, *n* = 20); “individuals with good adherence to ART” (100%, *n* = 20); “individuals with an optimistic attitude towards life and a positive attitude to HIV infection” (100%, *n* = 20); “individuals with a health condition” (100%, *n* = 20); “individuals who have been receiving ART for more than 6 months” (80%, *n* = 16). Furthermore, two items that gained “nearing” consensus, that is, reaching > 65% agreement, were “individuals with at least a high school diploma” (75%, *n* = 15), “individuals with no substance abuse during the past 3 years” (70%, *n* = 14), and “volunteers willing to disclose their infection status of HIV” (65%, *n* = 13).

Two items were excluded since they did not achieve a consensus level of 60%. Excluded items included “volunteers with a viral load below the detection limit” (55%, *n* = 11) and “volunteers with CD4 counts ≥ 200 cells/*μ*l” (40%, *n* = 8). In addition, the item “individuals with good adherence to ART” was also excluded from the second round of consultation because one panel believed this condition is naturally contained within the description of the item “individuals with good self-management skills for HIV infection.” Two new items were proposed including “volunteers with a stable and long term residence in Shanghai” and “volunteers who have a good social support” and were added to the questionnaire for the second round of consultation.

### 3.3. The Second Round

**Figure 1:**
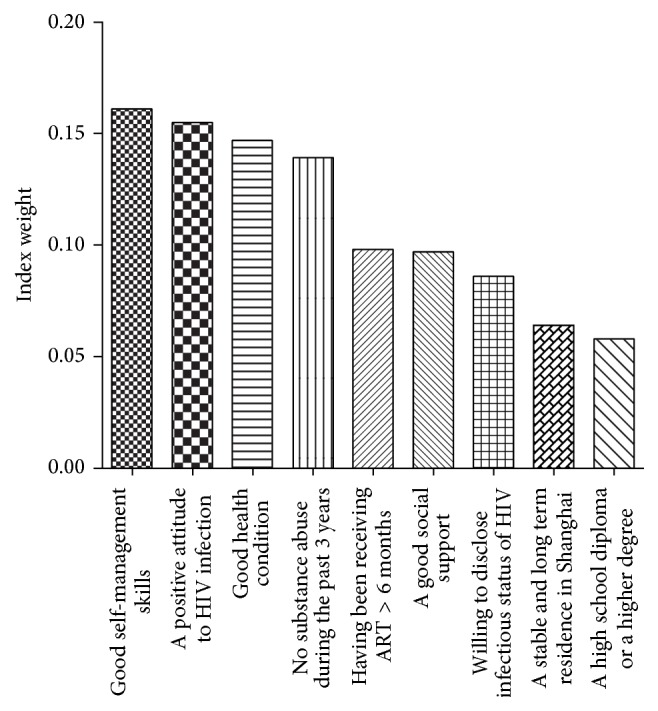
The index weight of each selected indicator.

The Precedence Chart Method was adopted in the second round to evaluate the index weight of the nine primary items generated by the assessment of the first round of consultation. As shown in Figure 1, “individuals with good self-management skills for HIV” (0.161) ranked first, followed by “individuals with an optimistic attitude towards life and a positive attitude to HIV infection” (0.155), “individuals with a health condition” (0.147), “individuals with no substance abuse during the past 3 years” (0.139), “individuals who have been receiving ART for more than 6 months” (0.098), “volunteers with a good social support” (0.097), “volunteers willing to disclose their infection status of HIV” (0.086), “volunteers with a stable and long term residence in Shanghai” (0.064), and “individuals with at least a high school diploma” (0.058). Kendall's W for concordance of the second round was 0.546, *χ* ^2^ = 20.64, *p* < 0.01, which indicates excellent agreement between the expert panelists. In conclusion, all the nine items reached a complete consensus.

## 4. Discussion

Peer education or peer support is believed to be a reasonable way to address the shortage of professional medical workforce in intervention of the spreading of HIV infection. Studies in the past decade have proved that peer education is effective in promoting safe sex behaviors and decreasing HIV/Sexually Transmitted Diseases (STD) incidence in the target group [17]. To initiate an HIV intervention peer education program, a clear criterion for the selection of peer educators needs to be developed in the first place. By adopting the Delphi method, a panel of experts including physicians and nurses with extensive working experiences in HIV treatment clinics were requested to share thoughts on norms or standards they deemed essential for recruiting HIV intervention peer educators.

In round I of the Delphi study, two of the initial ten items (e.g., “volunteers with no substance abuse during the past 3 years” and “volunteers willing to disclose their infection status of HIV”) did not gain a consensus level higher than 70%. Although a consensus level of 80% is believed to mark a clear majority opinion, these two items remained to be added into the questionnaire for the second round of consultation, as some studies demonstrated that both drug abuse and alcohol addiction are unfavorable factors for the treatment of HIV infection [4, 7]. Moreover, it was also found that injection drug users who had refrained from using drugs for more than 3 years mostly would not become drug addicts again [7]. The rationale for keeping “volunteers willing to disclose their infection status of HIV” on the questionnaire is that infected volunteers who are willing to disclose their status most likely will work respectfully with everyone and become an inspiration to infected peers or even high-risk individuals.

It is noteworthy that the rank order of the top three criteria for selecting peer educators was the same for both round I and round II consultations, which indicated these three criteria were vitally important. However, as pointed out by several panelists, the priority ranking may not represent a clear choice of one criterion over the others. It may be more useful to view them as complementary rather than competing. Actually, it is very reasonable to recruit “volunteers with a stable and long term residence in Shanghai” if we plan to implement the peer education system in Shanghai. With the same thinking, it may be most appropriate to select “volunteers with at least a high school diploma” to guarantee they could at least be able to receive an effective peer educator training.

Admittedly, our study has its limitation which is inherited from the Delphi method [18]. While the identified items or criteria are a useful starting point to initiate a general peer education program, the real context (i.e., men who have sex with men (MSM), injection drug user (IDU), or commercial sex workers) which peer educators will be working on has not been considered. Anyway, our study, for the first time, tried to provide an initial framework from which HIV/AIDS care providers can begin to recruit peers to construct the first peer education program in Shanghai or even in China. Now the challenge will be to select enough peer educators based on these criteria and see how they will work in AIDS care.

## 5. Conclusions

This study was performed to define criteria for selection of peer educators in HIV management that are suitable for current practice in China, and nine selection criteria were identified through Delphi method. These initial criteria provide a basis for the successful development of peer education. However, the reliability and feasibility of the indicators included in this study need to be further tested in real practice.

## Figures and Tables

**Table 1:** Participant characteristics.

Variables	*n* (%)
*Sex*	
Male	7 (35)
Female	13 (65)
*Age (years)*	
20–30	4 (20)
30–40	7 (35)
40–50	9 (45)
*Work experience in HIV (years)*	
<10	3 (15)
10–20	14 (70)
>20	3 (15)
*Occupation*	
Physician	9 (45)
Nurse	11 (55)
*Highest degree*	
Ph.D. or M.D.	6 (30)
Master' degree	6 (30)
Bachelor's degree	7 (35)
High school or lower	1 (5)
*Job title*	
Chief Physician	1 (5)
Deputy Chief Physician	3 (15)
Attending Physician	5 (25)
Chief Nurse	2 (10)
Nurse in Charge	9 (45)
